# Supplementary material for: Dynalign II: common secondary structure prediction for RNA homologs with domain insertions
Source: Nucleic Acids Res. 2014 Nov 21;42(22):13939–48. doi: 10.1093/nar/gku1172 (PMC4267632; doi:10.1093/nar/gku1172)
Supplement: SUPPLEMENTARY DATA [file supp_gku1172_nar-02021-z-2014-File012.zip › manual/RNA_class/html/class_multifind__object.html]

RNAstructure Classes: Multifind\_object Class Reference

- Main Page
- Classes
- Files

- Class List
- Class Hierarchy
- Class Members

# Multifind\_object Class Reference

Multifind\_object Class.
More...

`#include <Multifind_object.h>`

Inherits Multilign\_object.

List of all members.

|  |  |
| --- | --- |
|  |  |
| Public Member Functions | |
|  | Multifind\_object (const string &outputmultifind, const vector< string > &ctfiles, const vector< string > &inputalignment, const vector< string > &inputsequences, const int &processors, TProgressDialog \*progress=NULL) |
| int | Multifind\_Predict () |
|  | The core function doing Multilign calculation and SVM prediction. |
| Private Member Functions | |
| double | sum\_multifind (vector< double > &series) |
| double | average\_multifind (vector< double > &series) |
| double | variation\_multifind (vector< double > &series) |
| double | sum\_multifind (vector< float > &series) |
| double | average\_multifind (vector< float > &series) |
| double | variation\_multifind (vector< float > &series) |
| double | sum\_multifind (vector< int > &series) |
| double | average\_multifind (vector< int > &series) |
| double | variation\_multifind (vector< int > &series) |
| double | normalized\_ensemble\_defect (RNA \*rna) |
| double | entropy (vector< char > column) |
| double | average\_entropy () |
| vector< double > | single\_z\_predict (string sequence, svm\_model \*model\_folding\_average, svm\_model \*model\_folding\_std, svm\_model \*model\_ensemble\_average, svm\_model \*model\_ensemble\_std) |
| int | get\_gap (string sequence) |
| double | common\_energies () |
| vector< double > | predict\_ncRNA\_probabilities (double sci, double entropy, double single\_z, double ensemble\_defect\_z) |
| Private Attributes | |
| int | num\_processors |

---

## Detailed Description

Multifind\_object Class.

The Multifind\_object class provides an entry point for the Multifind algorithm. 

---

## Constructor & Destructor Documentation

|  |  |  |  |  |
| --- | --- | --- | --- | --- |
| Multifind\_object::Multifind\_object | ( | const string & | *outputmultifind*, |  |
|  |  | const vector< string > & | *ctfiles*, |  |
|  |  | const vector< string > & | *inputalignment*, |  |
|  |  | const vector< string > & | *inputsequences*, |  |
|  |  | const int & | *processors*, |  |
|  |  | TProgressDialog \* | *progress* = `NULL` |  |
|  | ) |  |  |  |

Constructor:

**Parameters:**
:   |  |  |  |
    | --- | --- | --- |
    |  | *outputmultifind* | is the name of the Multifind output file to which the output is written to. |
    |  | *ctfiles* | is a vector of strings storing the names of the ct files to which the output structures are written to. |
    |  | *inputalignment* | is a vector of strings storing the input sequences in the alignment (with gaps). |
    |  | *inputsequences* | is a vector of strings storing the input sequences in the alignment (without gaps). |
    |  | *processors* | is a interger indicating the number of processors required by Multifind in smp calculations.(only applicable in smp version) |
    |  | *progress* | is a TProgressDialog for reporting progress of the calculation to the user. The default value of NULL means that no communication is provided. |

---

## Member Function Documentation

|  |  |  |  |  |
| --- | --- | --- | --- | --- |
| double Multifind\_object::average\_entropy | ( |  | ) | `[private]` |

|  |  |  |  |  |  |
| --- | --- | --- | --- | --- | --- |
| double Multifind\_object::average\_multifind | ( | vector< int > & | *series* | ) | `[private]` |

|  |  |  |  |  |  |
| --- | --- | --- | --- | --- | --- |
| double Multifind\_object::average\_multifind | ( | vector< float > & | *series* | ) | `[private]` |

|  |  |  |  |  |  |
| --- | --- | --- | --- | --- | --- |
| double Multifind\_object::average\_multifind | ( | vector< double > & | *series* | ) | `[private]` |

|  |  |  |  |  |
| --- | --- | --- | --- | --- |
| double Multifind\_object::common\_energies | ( |  | ) | `[private]` |

|  |  |  |  |  |  |
| --- | --- | --- | --- | --- | --- |
| double Multifind\_object::entropy | ( | vector< char > | *column* | ) | `[private]` |

|  |  |  |  |  |  |
| --- | --- | --- | --- | --- | --- |
| int Multifind\_object::get\_gap | ( | string | *sequence* | ) | `[private]` |

|  |  |  |  |  |
| --- | --- | --- | --- | --- |
| int Multifind\_object::Multifind\_Predict | ( |  | ) |  |

The core function doing Multilign calculation and SVM prediction.

|  |  |  |  |  |  |
| --- | --- | --- | --- | --- | --- |
| double Multifind\_object::normalized\_ensemble\_defect | ( | RNA \* | *rna* | ) | `[private]` |

|  |  |  |  |  |
| --- | --- | --- | --- | --- |
| vector< double > Multifind\_object::predict\_ncRNA\_probabilities | ( | double | *sci*, |  |
|  |  | double | *entropy*, |  |
|  |  | double | *single\_z*, |  |
|  |  | double | *ensemble\_defect\_z* |  |
|  | ) |  |  | `[private]` |

|  |  |  |  |  |
| --- | --- | --- | --- | --- |
| vector< double > Multifind\_object::single\_z\_predict | ( | string | *sequence*, |  |
|  |  | svm\_model \* | *model\_folding\_average*, |  |
|  |  | svm\_model \* | *model\_folding\_std*, |  |
|  |  | svm\_model \* | *model\_ensemble\_average*, |  |
|  |  | svm\_model \* | *model\_ensemble\_std* |  |
|  | ) |  |  | `[private]` |

|  |  |  |  |  |  |
| --- | --- | --- | --- | --- | --- |
| double Multifind\_object::sum\_multifind | ( | vector< int > & | *series* | ) | `[private]` |

|  |  |  |  |  |  |
| --- | --- | --- | --- | --- | --- |
| double Multifind\_object::sum\_multifind | ( | vector< float > & | *series* | ) | `[private]` |

|  |  |  |  |  |  |
| --- | --- | --- | --- | --- | --- |
| double Multifind\_object::sum\_multifind | ( | vector< double > & | *series* | ) | `[private]` |

|  |  |  |  |  |  |
| --- | --- | --- | --- | --- | --- |
| double Multifind\_object::variation\_multifind | ( | vector< int > & | *series* | ) | `[private]` |

|  |  |  |  |  |  |
| --- | --- | --- | --- | --- | --- |
| double Multifind\_object::variation\_multifind | ( | vector< float > & | *series* | ) | `[private]` |

|  |  |  |  |  |  |
| --- | --- | --- | --- | --- | --- |
| double Multifind\_object::variation\_multifind | ( | vector< double > & | *series* | ) | `[private]` |

---

## Member Data Documentation

int Multifind\_object::num\_processors `[private]`

---

The documentation for this class was generated from the following files:

- F:/RNAstructure-co-from-repository/RNAstructure/RNA\_class/Multifind\_object.h- F:/RNAstructure-co-from-repository/RNAstructure/RNA\_class/Multifind\_object.cpp

---

Generated on Tue Jul 30 10:52:29 2013 for RNAstructure Classes by 
 1.5.7.1
